# Pre-operative circulating tumor cells predict worse treatment outcome in patients with high-grade serous ovarian cancer

**DOI:** 10.1038/s41388-026-03747-3

**Published:** 2026-04-04

**Authors:** Annabelle Lobermeyer, Jenna Sophie Schöllhorn, Paul N. Rademacher, Maximilian C. Wankner, Sandra Lenz, Cornelia Coith, Piet Sonnemann, Anna Jaeger, Leticia Oliveira-Ferrer, Linn Woelber, Sabine Riethdorf, Klaus Pantel, Barbara Schmalfeldt, Mina Netkova-Heintzen, Katharina Prieske, Simon A. Joosse

**Affiliations:** 1 Department of Tumor Biology, University Medical Center Hamburg-Eppendorf, Hamburg, Germany; 2 Mildred Scheel Career Center (MSNZ Hamburg), University Medical Center Hamburg-Eppendorf, Hamburg, Germany; 3 Department of Oncology, Hematology and Bone Marrow Transplantation with Section Pneumology, Department of Internal Medicine II, University Medical Center Hamburg-Eppendorf, Hamburg, Germany; 4 Department of Gynecology, University Medical Center Hamburg-Eppendorf, Hamburg, Germany

**Keywords:** Ovarian cancer, Tumour biomarkers, Predictive markers, Prognostic markers

## Abstract

High-grade serous ovarian cancer (HGSOC) is the most lethal gynecological malignancy. Pre- and post-operative, non-invasive biomarkers for reliable treatment outcome prediction, (minimal) residual disease detection, and prognosis are not yet established in clinical practice for these patients. This prospective study quantified circulating tumor cells (CTCs) in 7.5 ml peripheral blood in 56 women with FIGO stages IIIC and IV HGSOC before and after primary cytoreductive surgery and in 9 women with benign ovarian disease. Clinical outcomes were assessed during the median follow-up of 35.4 months. CTCs were detected in 48.2% (27/56) of patients pre-operatively and in 46.4% (26/56) post-operatively, but not in benign controls. Pre-operative CTCs were associated with suboptimal cytoreductive surgery (OR = 15.6, 95% CI: 2.97–127.0, *p* = 0.0031), worse platinum response (*p* = 0.0173), lymph node metastases (*p* = 0.0151), and shorter progression-free (*p* = 0.0045) and overall survival (*p* = 0.0241). Post-operative CTC-augmented residual tumor was significantly associated with worse OS (*p* = 0.047). In multivariable analyses, pre-operative CTCs remained an independent surrogate marker for incomplete debulking, platinum resistance, and poor survival in HGSOC. Therefore, the quantification of CTCs in HGSOC pre- and post-operatively may be used to guide treatment selection, reduce surgical morbidity, improve the healthcare provider’s resource allocation and planning, and enhance patient counseling.

## Introduction

High-grade serous ovarian cancer (HGSOC) is the most lethal gynecological malignancy affecting women worldwide [1]. The expected 5-year survival rate ranges from 26 to 48%, largely due to frequent diagnosis at an advanced stage (International Federation of Gynecology and Obstetrics (FIGO) stages III–IV), a delay attributed to the lack of specific early symptoms and effective screening methods [2, 3]. Currently, the highest response rate to treatment is achieved by surgery followed by platinum-based chemotherapy. The main goal of primary cytoreductive (debulking) surgery is to attain a macroscopic disease-free state [4, 5]; however, both patients and clinicians are faced with numerous challenges in practice. While suspicious lymph nodes are typically excised to prevent tumor spread, systematic lymphadenectomy has not demonstrated a survival benefit [6, 7]. Similarly, neoadjuvant chemotherapy to reduce the tumor load fails to provide an advantage over upfront primary debulking surgery and adjuvant chemotherapy proves ineffective in 20–30% of HGSOC patients due to platinum resistance [8]. In addition, extensive debulking surgery carries a high-risk of post-operative complications, such as infection, prolonged recovery, fatigue, pain, digestive issues, emotional stress, and secondary comorbidities [9–12]. As a result, accomplishing a disease-free state by surgery is frequently unattainable and the majority of patients relapse within 2-3 years of their initial diagnosis [13, 14]. Together, these limitations highlight the persistent challenges in improving long-term outcomes for women with advanced ovarian cancer.

Identifying patients with high-risk of incomplete tumor resection, lymph node involvement, and platinum sensitivity before surgery could help to predict neoadjuvant chemotherapy benefit, reduce surgical morbidity by avoiding aggressive surgery in poor outcome patients, improve the healthcare provider’s resource allocation and planning, and enhance patient counseling. However, no radiologic or antigen-based screening method currently exists to reliably predict therapy outcome. Serum cancer antigen 125 (CA125) can be a strong risk factor of suboptimal cytoreduction, especially in combination with other biomarkers, but lacks the ability to predict optimal cytoreduction accurately with sensitivities and specificities ranging between 49–78% and 59–77%, respectively [15–17]. Consequently, blood-based liquid biopsy techniques, including the detection of circulating tumor cells (CTCs), have gained much attention for their potential high sensitivity and specificity [18]. Although CTCs in ovarian cancer patients have prognostic value in terms of PFS and OS [19], there is no clear evidence for the predictive value of CTCs in terms of treatment outcome. Liquid biopsy, including cell-free DNA, has previously been linked to predicting therapy resistance [20–24], but there is only restricted information available for CTCs enriched with CellSearch System [25].

In this study, we analyzed the presence of CTCs in advanced HGSOC patients to evaluate their predictive value for achieving a complete tumor resection with primary debulking surgery, chemosensitivity, and their prognostic value after surgery.

## Materials/subjects and methods

### Study population and clinical follow-up

Patients eligible for this study were women (≥18 years old) newly diagnosed with HGSOC (FIGO IIIC-IV). Between July 2020 and September 2024, 186 women with suspected pelvic masses were recruited. Of these, 79 scheduled for ovarian cancer surgery at the University Medical Center Hamburg–Eppendorf were confirmed as HGSOC FIGO IIIC–IV; 23 were lost to follow-up. The final study included 56 advanced HGSOC patients and 9 women with a benign diagnosis. This study was approved by the local ethical board (Hamburger Ärztekammer, number PV5392-3704_6-BO-ff) and written informed consent was obtained from all patients. This study was conducted in accordance with the Declaration of Helsinki, local ethical guidelines, and regulatory requirements. After initial debulking surgery, residual tumor was defined as the standard clinical measure of remaining disease categorized as R0 (no visible lesions), R1 (lesion ≤1 cm), or R2 (lesion >1 cm). Information on whether lymph nodes were resected during surgery and whether they were tumor-infiltrated was obtained exclusively from pathology reports. All patients received 6 cycles of adjuvant chemotherapy with carboplatin and paclitaxel in combination with bevacizumab, followed by maintenance therapy with bevacizumab and olaparib or niraparib until disease recurrence according to local guidelines. Patients were categorized into four subgroups based on the platinum-free interval (PFI), defined as the time from the last platinum dose to documented disease relapse according to the Gynecologic Cancer InterGroup (GCIG) consensus: platinum-refractory (PFI ≤ 1 month), platinum-resistant (1 month < PFI ≤ 6 months), partially platinum-sensitive (6 months < PFI ≤ 12 months), and platinum-sensitive (PFI > 12 months) [26].

### Sample processing

Within 24 h before surgery, 7.5 ml of peripheral blood was obtained from the antecubital vein or central venous catheter in K3 EDTA-containing tubes (Sarstedt, Cat. No. 01.1605.001). Blood was transferred to CellSave preservative tubes (Menarini Silicon Biosystems, Cat. No. 7900005) within 4 h and processed within 96 h. Blood was obtained again within 2–3 days after surgery or upon leaving the post-anesthetic care unit. CTC enrichment and identification were performed using the CellSearch System per manufacturer protocol (www.cellsearchctc.com). Briefly, the system enriches CTCs via immunomagnetic ferrofluid nanoparticles coated with anti-epithelial cell adhesion molecule (EpCAM) antibody, followed by immunofluorescence staining with anti-keratin (PE = phycoerythrin) and anti-CD45 antibodies (APC = allophycocyanin). CTCs (keratin^+^/DAPI^+^ and CD45^−^ cells) were identified using the Celltracks Analyzer II.

### Definition of CTC threshold

The initial studies with CellSearch System have shown that ≥5 CTCs/7.5 ml peripheral blood is correlated with poor prognosis in metastatic breast, colorectal, and prostate cancer [27–29]. Previous CellSearch-based studies correlated primary advanced ovarian cancer prognosis to CTC cut-offs of ≥2 CTCs/7.5 ml [30, 31]. Compared to advanced breast, prostate, and colorectal cancer, patients with primary ovarian cancer have fewer detectable CTCs in general, indicating that a lower cut-off is more appropriate [30–34]. Therefore, we applied two thresholds to correlate CTC status with clinical outcome: 0 vs. ≥1 CTCs, termed CTC negative vs. positive; and <2 vs. ≥2 CTCs, termed CTC low vs. high. A threshold of at least two CTCs eliminates potential false-positive CellSearch System counts such as rare circulating epithelial cells found in healthy individuals [35, 36].

### Statistical analysis

All analyses were performed in R version 4.2.1 and In-Silico Online v2.4.0 [37]. Overall survival (OS) was defined as the time from primary debulking surgery to death from any cause or censored at last contact, and PFS as the time from surgery to progression or relapse. Continuous variables were summarized by mean and range and analyzed using t-test. Categorical variables were listed as frequencies and percentages and associations were calculated using Fisher’s exact test. Survival analyses were performed using log-rank test and Cox proportional hazards regression model. To assess potential associations between CTC spread and anatomical locations of tumor growth, point-biserial correlations followed by correction for multiple testing were employed. Both multivariable and multivariate analyses were performed using generalized linear models, the latter with multivariate extension (R package mvabund, version 4.2.1). An (adjusted) *p*-value of less than 0.05 was considered statistically significant.

## Results

### Patient characteristics

56 women newly diagnosed with HGSOC and 9 women with benign findings were enrolled. The median age of patients at the time of initial diagnosis was 64.4 years (range: 36–84 years) and the median follow-up period was 35.4 months (range: 1–53.9 months). 37 patients (66.1%) were diagnosed with FIGO stage IIIC and 19 patients (33.9%) were diagnosed with FIGO stage IV. Optimal debulking with complete macroscopic tumor resection (R0) was achieved in 18 cases (32.1%). Residual tumor of ≤1 cm (R1) remained in 24 patients (42.9%) whereas residual tumor >1 cm (R2) remained in 14 patients (25.0%). A total of 34 patients (60.7%) developed a progression or recurrence within a median time from initial diagnosis of 20.6 months (range: 1–43.6 months). 21 patients (37.5%) died within the study period. Post-operative residual tumor was correlated with worse PFS (*p* = 0.0413; Supplementary Fig. 1A), but not OS (*p* = 0.0691; Supplementary Fig. 1B). Patients diagnosed with R2 had significantly increased risk of progression in comparison with patients with R0 (HR = 3.40, 95% CI: 1.29–8.98, *p* = 0.0134).

One or more and ≥2 CTCs were detected in 48.21% (27/56) and 26.79% (15/56) of patients pre-operatively and 46.43% (26/56) and 19.64% (11/56) post-operatively, respectively (Table 1). Median pre- and post-operative CTC counts of CTC-positive cases were 2 (range: 1–30) and 1 (range: 1–20).

**Table 1:** Patient demographics.

A												
	Pre-operative						Post-operative					
	<1 CTC (*n* = 29)	≥1 CTC (*n* = 27)	*p-value*	<2 CTC (*n* = 41)	≥2 CTC (*n* = 15)	*p-value*	<1 CTC (*n* = 30)	≥1 CTC (*n* = 26)	*p-value*	<2 CTC (*n* = 45)	≥2 CTC (*n* = 11)	*p-value*
**Age**^a^			0.9348			0.0934			0.0057			0.7245
Mean	64	64		65	60		61	68		64	65	
**FIGO**^b^			0.7789			0.3392			>0.9999			0.1563
IIIC	20 (69.0%)	17 (63.0%)		29 (70.7%)	8 (53.3%)		20 (66.7%)	17 (65.4%)		32 (71.1%)	5 (45.5%)	
IV	9 (31.0%)	10 (37.0%)		12 (29.3%)	7 (46.7%)		10 (33.3%)	9 (34.6%)		13 (28.9%)	6 (54.5%)	
**T stage**^b^			>0.9999			>0.9999			0.4935			>0.9999
T2	1 (3.4%)	1 (3.7%)		2 (4.9%)	0 (0.0%)		2 (6.7%)	0 (0.0%)		2 (4.4%)	0 (0.0%)	
T3	28 (96.6%)	26 (96.3%)		39 (95.1%)	15 (100.0%)		28 (93.3%)	26 (100.0%)		43 (95.6%)	11 (100.0%)	
**N stage**^b^			0.1582			0.4157			0.4587			>0.9999
N0	10 (55.6%)	4 (26.7%)		12 (48.0%)	2 (25.0%)		8 (36.4%)	6 (54.5%)		11 (40.7%)	3 (50.0%)	
N1	8 (44.4%)	11 (73.3%)		13 (52.0%)	6 (75.0%)		14 (63.6%)	5 (45.5%)		16 (59.3%)	3 (50.0%)	
**V stage**^b^			0.1908			0.3816			>0.9999			0.3507
V0	29 (100.0%)	21 (91.3%)		40 (97.6%)	10 (90.9%)		27 (96.4%)	23 (95.8%)		41 (97.6%)	9 (90.0%)	
V1	0 (0.0%)	2 (8.7%)		1 (2.4%)	1 (9.1%)		1 (3.6%)	1 (4.2%)		1 (2.4%)	1 (10.0%)	
**L stage**^b^			>0.9999			>0.9999			>0.9999			>0.9999
L0	12 (41.4%)	11 (44.0%)		18 (43.9%)	5 (38.5%)		12 (41.4%)	11 (44.0%)		18 (41.9%)	5 (45.5%)	
L1	17 (58.6%)	14 (56.0%)		23 (56.1%)	8 (61.5%)		17 (58.6%)	14 (56.0%)		25 (58.1%)	6 (54.5%)	
**Pn stage**^b^			>0.9999			>0.9999			>0.9999			>0.9999
Pn0	26 (96.3%)	19 (100.0%)		36 (97.3%)	9 (100.0%)		23 (95.8%)	22 (100.0%)		36 (97.3%)	9 (100%)	
Pn1	1 (3.7%)	0 (0.0%)		1 (2.7%)	0 (0.0%)		1 (4.2%)	0 (0.0%)		1 (2.7%)	0 (0.0%)	
**Residual tumor status**^b^			0.0031			0.0209			0.0361			0.5752
R0	13 (44.8%)	5 (18.5%)		15 (37.5%)	3 (21.4%)		14 (46.7%)	4 (15.4%)		16 (35.6%)	2 (18.2%)	
R1	14 (48.3%)	10 (37.0%)		20 (48.8%)	4 (26.7%)		9 (30.0%)	15 (57.7%)		18 (40%)	6 (54.5%)	
R2	2 (6.9%)	12 (44.4%)		6 (15.0%)	8 (57.1%)		7 (23.3%)	7 (26.9%)		11 (24.4%)	3 (27.3%)	
**Pre-operative CA125 [U/ml]**^b^			>0.9999			0.1713						
<385	7 (30.4%)	8 (32.0%)		13 (38.2%)	2 (14.3%)							
≥385	16 (69.6%)	17 (68.0%)		21 (61.8%)	12 (85.7%)							
**Pre-operative CA125 [U/ml]**^b^			>0.9999			0.2135						
<500	10 (43.5%)	11 (44.0%)		17 (50.0%)	4 (28.6%)							
≥500	13 (56.5%)	14 (56.0%)		17 (50.0%)	10 (71.4%)							

B										
	Surgery dynamics: ≥1 CTC threshold					Surgery dynamics: ≥2 CTC threshold				
	Persistent negative (*n* = 16)	Decrease (*n* = 14)	Increase (*n* = 13)	Persistent positive (*n* = 13)	*p-value*	Persistent negative (*n* = 36)	Decrease (*n* = 9)	Increase (*n* = 5)	Persistent positive (*n* = 6)	*p-value*
**Age**^a^					0.0500					0.0561
Mean	60	61	69	67		66	56	63	67	
**FIGO**^b^					0.9807					0.2394
IIIC	11 (68.8%)	9 (64.3%)	9 (69.2%)	8 (61.5%)		27 (75.0%)	5 (55.6%)	2 (40.0%)	3 (50.0%)	
IV	5 (31.2%)	5 (35.7%)	4 (30.8%)	5 (38.5%)		9 (25.0%)	4 (44.4%)	3 (60.0%)	3 (50.0%)	
**T stage**^b^					>0.9999					>0.9999
T2	1 (6.2%)	1 (7.1%)	0 (0.0%)	0 (0.0%)		2 (5.6%)	0 (0.0%)	0 (0.0%)	0 (0.0%)	
T3	15 (93.8%)	13 (92.9%)	13 (100.0%)	13 (100.0%)		34 (94.4%)	9 (100.0%)	5 (100.0%)	6 (100.0%)	
**N stage**^b^					0.1399					0.1037
N0	5 (41.7%)	3 (30.0%)	5 (83.3%)	1 (20.0%)		9 (40.9%)	2 (40.0%)	3 (100.0%)	0 (0.0%)	
N1	7 (58.3%)	7 (70.0%)	1 (16.7%)	4 (80.0%)		13 (59.1%)	3 (60.0%)	0 (0.0%)	3 (100.0%)	
**V stage**^b^					0.3401					0.3620
V0	16 (100.0%)	11 (91.7%)	13 (100.0%)	10 (90.9%)		35 (97.2%)	6 (100.0%)	5 (100.0%)	4 (80.0%)	
V1	0 (0.0%)	1 (8.3%)	0 (0.0%)	1 (9.1%)		1 (2.8%)	0 (0.0%)	0 (0.0%)	1 (20.0%)	
**L stage**^b^					0.9631					0.9005
L0	6 (37.5%)	6 (46.2%)	6 (46.2%)	5 (41.7%)		15 (41.7%)	3 (42.9%)	3 (60.0%)	2 (33.3%)	
L1	10 (62.5%)	7 (53.8%)	7 (53.8%)	7 (58.3%)		21 (58.3%)	4 (57.1%)	2 (40.0%)	4 (66.7%)	
**Pn stage**^b^					>0.9999					>0.9999
Pn0	13 (92.9%)	10 (100.0%)	13 (100.0%)	9 (100.0%)		31 (96.9%)	5 (100.0%)	5 (100.0%)	4 (100.0%)	
Pn1	1 (7.1%)	0 (0.0%)	0 (0.0%)	0 (0.0%)		1 (3.1%)	0 (0.0%)	0 (0.0%)	0 (0.0%)	
**Residual tumor status** ^b^					0.0022					0.0507
R0	10 (62.5%)	4 (28.6%)	3 (23.1%)	1 (7.7%)		14 (38.9%)	2 (22.2%)	1 (20.0%)	1 (16.7%)	
R1	6 (37.5%)	3 (21.4%)	8 (61.5%)	7 (53.8%)		17 (47.2%)	1 (11.1%)	3 (60.0%)	3 (50.0%)	
R2	0 (0.0%)	7 (50.0%)	2 (15.4%)	5 (38.5%)		5 (13.9%)	6 (66.7%)	1 (20.0%)	2 (33.3%)	
**Pre-operative CA125 [U/ml]**^b^					0.3367					0.0849
<385	4 (33.3%)	6 (50.0%)	3 (27.3%)	2 (15.4%)		13 (43.3%)	2 (25.0%)	0 (0.0%)	0 (0.0%)	
≥385	8 (66.7%)	6 (50.0%)	8 (72.7%)	11 (84.6%)		17 (56.7%)	6 (75.0%)	4 (100.0%)	6 (100.0%)	
**Pre-operative CA125 [U/ml]**^b^					0.5453					0.0758
<500	6 (50.0%)	4 (36.4%)	7 (58.3%)	4 (30.8%)		17 (56.7%)	0 (0.0%)	3 (37.5%)	1 (16.7%)	
≥500	6 (50.0%)	7 (63.6%)	5 (41.7%)	9 (69.2%)		13 (43.3%)	4 (100.0%)	5 (62.5%)	5 (83.3%)	

Summary of clinical and pathological characteristics stratified by circulating tumor cell (CTC) burden and CTC dynamics before and after debulking surgery. **A**) CTC counts were analyzed using two thresholds (≥1 and ≥2 CTCs) separately for pre- and post-operative time points. **B**) Additionally, patients were categorized by CTC dynamics across timepoint using the same threshold.

Persistent negative=CTCs below threshold pre- and post-operatively; Decrease=CTCs above threshold pre- and below threshold post-operatively; Increase=CTCs below threshold pre- and above threshold post-operatively; Persistent positive=CTCs above threshold at both time points.

FIGO=International Federation of Gynecology and Obstetrics, T=Tumor, N=Nodes, V=Venous invasion, L=Lymphatic invasion, Pn=Perineural invasion, CA125=Cancer antigen 125

^a^ ANOVA, ^b^ Fisher's Exact Test

### Pre-operative CTCs are predictive of surgery outcome and platinum response

CTCs pre-operatively, as well as the increase from 0 to at least 1 CTC post-operatively was significantly correlated with residual tumor, whereas the initial and persistent absence of CTCs pre- and post-operatively was associated with optimal tumor removal (R0) during debulking surgery (Table 1). Among patients with R2 resection status, 44.4% and 57.1% had ≥1 and ≥2 CTCs pre-operatively, compared to 18.5% and 21.4% among patients with R0 status (*p* = 0.0031 and *p* = 0.0209, respectively), resulting in a 15.6- (95% CI: 2.97–127.0, *p* = 0.0031) and 6.67-times higher odds ratio (95% CI: 1.42–39.5, *p* = 0.0225) of having an R2 residual tumor compared to those with fewer CTCs. In multivariable analysis, CTC status was significantly associated with increasing R-status (*p* = 0.0188), as was FIGO stage IV (*p* = 0.0229), and age (*p* = 0.0180), whereas CA125 concentration was not (*p* = 0.5408; Table 2). Taken together, cytoreductive surgery (R0) was predicted with 57% and 32% sensitivity, 72% and 83% specificity at ≤1 and ≤2 CTCs pre-operatively, respectively, and vice versa for suboptimal cytoreduction (R1/R2). CA125 at <385 or <500 U/ml predicted R0 with 64% and 52% sensitivity and 20% and 33% specificity. Pre-operative CA125 in combination with pre-operative CTCs negative status resulted in an AUC of 0.65 for predicting R0, whereas pre-operative CA125 in combination with <2 pre-operative CTCs resulted in an AUC of 0.56.

**Table 2:** Multivariable analyses for therapy outcome.

R-status	Univariable analysis			Multivariable analysis		
Covariate	Odds ratio	95% CI	*p*-value	Odds ratio	95% CI	*p*-value
CTC ≥ 2	4.539	(1.35–15.261)	0.0145	5.585	(1.33–23.444)	0.0188
Age	1.050	(0.996–1.106)	0.0678	1.086	(1.014–1.163)	0.0180
FIGO stage	2.375	(0.842–6.703)	0.1022	4.538	(1.233–16.701)	0.0229
CA125 ≥ 385 U/ml	0.626	(0.204–1.925)	0.4137	0.669	(0.184–2.427)	0.5408

Platinum resistance	Univariable analysis			Multivariable analysis		
Covariate	Odds ratio	95% CI	*p*-value	Odds ratio	95% CI	*p*-value
CTC ≥ 2	8.667	(1.632–55.43)	0.0140	12.013	(1.781–123.8)	0.0173
Age	1.028	(0.944–1.124)	0.5250	1.065	(0.964–1.205)	0.2480
FIGO stage	2.057	(0.353–10.77)	0.3946	2.127	(0.246–19.068)	0.4816
CA125 ≥ 385 U/ml	0.937	(0.183–5.438)	0.9388	0.885	(0.088–9.089)	0.9133

Odds ratios for increasing R-status and platinum resistance, using ordinal logistic regression and generalized linear models, respectively. Calculated are the 95% confidence interval (CI) of the odds ratio and *p*-value in uni- and multivariable analyses for the predictive variables CTC status pre-surgery, age, FIGO stage (reference: FIGO IIIC) and CA125 concentrations pre-operatively.

Platinum sensitivity (PFI > 12 months) was observed in 31/47 patients (66%), whereas 34% of patients were either partially sensitive (*n* = 8), resistant (*n* = 4), or refractory (*n* = 4); 9/56 cases were indeterminable due to the short follow-up and were therefore not considered. A high pre-operative CTC count (≥2 CTCs) was significantly associated with platinum resistance or refractory compared to (partial) sensitivity in uni- (*p* = 0.0140) and multivariable analysis (*p* = 0.0173), whereas age, FIGO stage, or pre-surgery CA125 were not informative in predicting platinum resistance (Table 2). CTC positivity of only ≥1 CTCs was not associated with either resistance or sensitivity to platinum-based chemotherapy.

In multivariate analysis, where R-status and platinum response were both included as treatment outcomes and CTC status and CA125 as dependent variables, both ≥1 (Deviance=2.94, *p* = 0.002) and ≥2 CTCs (Deviance=2.912, *p* = 0.002) were significantly associated with poor treatment outcome, whereas CA125 was not (Deviance=0.276, *p* = 0.459).

### Pre-operative CTCs are prognostic for worse PFS and OS

**Fig. 1:**
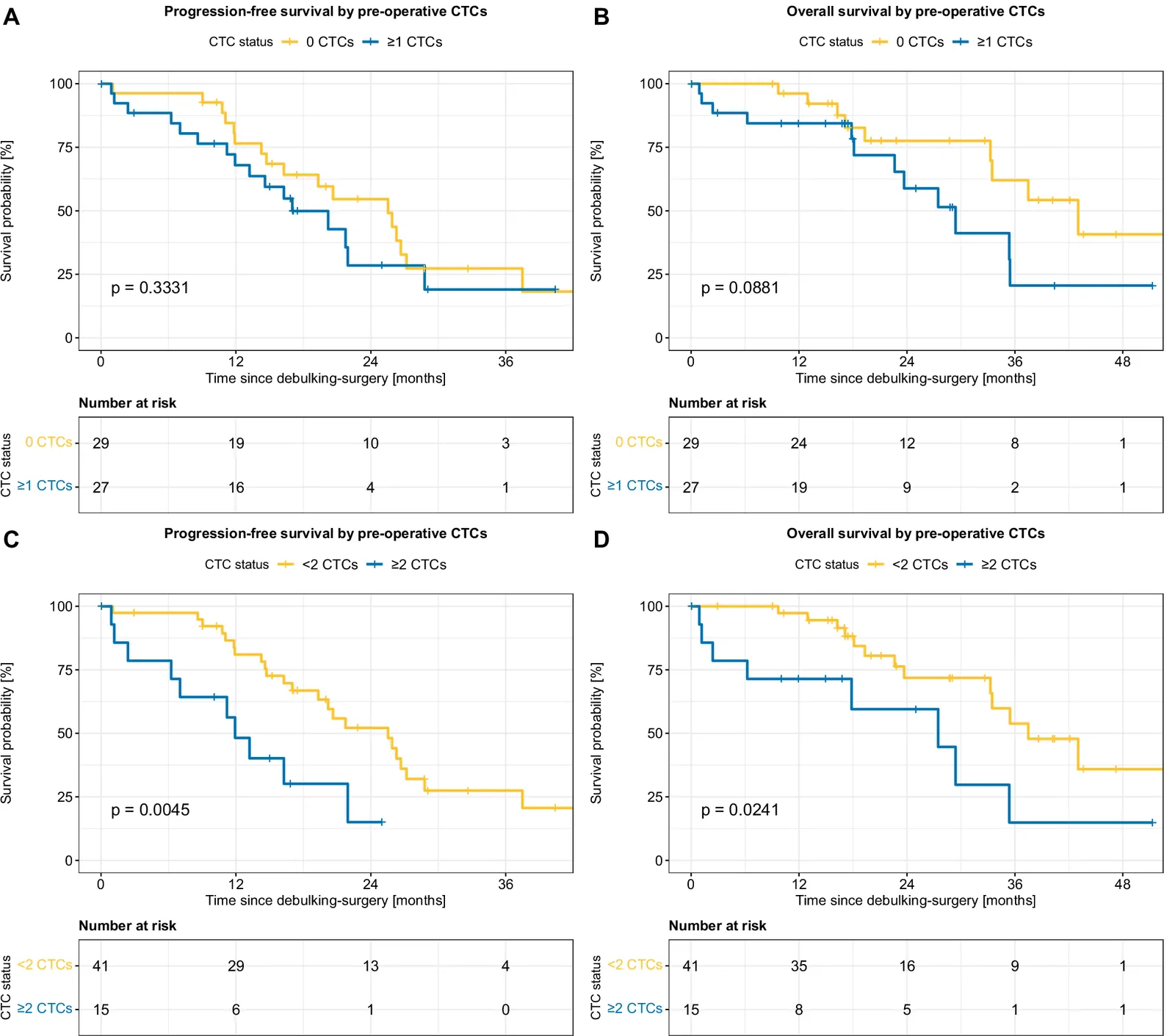
Progression-free and overall survival of HGSOC patients stratified based on the detection of ≥1 pre-operative CTCs (**A**, **B**) and ≥2 pre-operative CTCs (**C**, **D**).

One or more CTCs pre-operatively were not correlated with PFS (median: 25.6 vs. 16.9 months, *p* = 0.3331; Fig. 1A) or OS (median: 42.9 vs. 29.4 months, *p* = 0.0881; Fig. 1B). The presence of ≥2 CTCs pre-operatively was significantly correlated with worse PFS (median: 25.4 vs. 11.8 months, *p* = 0.0045; Fig. 1C) and worse OS (median: 37.5 vs. 27.5 months; *p* = 0.0241; Fig. 1D). The latter group was found to be independently associated to a 4.1-times higher risk of disease progression (*p* = 0.0077) and 3.5-times higher risk of earlier death (*p* = 0.0445) in univariable and multivariable analysis (Table 3). All other variables tested (age, CA125, R-status, and FIGO stage) were not independently correlated with PFS or OS.

**Table 3:** Multivariable survival analyses.

PFS	Univariable analysis			Multivariable analysis		
Covariate	Hazard ratio	95% CI	*p*-value	Hazard ratio	95% CI	p-value
CTC ≥ 2	3.042	(1.363–6.791)	0.0066	4.117	(1.453–11.662)	0.0077
Age	1.003	(0.967–1.040)	0.8654	1.030	(0.981–1.081)	0.2376
R-status	2.309	(1.057–5.041)	0.0357	1.793	(0.614–5.236)	0.2859
FIGO stage	1.585	(0.788–3.189)	0.1961	1.557	(0.609–3.976)	0.3550
CA125 ≥ 385 U/ml	1.000	(0.999–1.000)	0.4411	0.999	(0.999–1.000)	0.8280

OS	Univariable analysis			Multivariable analysis		
Covariate	Hazard ratio	95% CI	*p*-value	Hazard ratio	95% CI	*p*-value
CTC ≥ 2	2.677	(1.1–6.515)	0.0300	3.540	(1.032–12.148)	0.0445
Age	1.028	(0.982–1.077)	0.2362	1.037	(0.97–1.108)	0.2827
R-status	2.978	(0.989–8.966)	0.0523	1.577	(0.382–6.505)	0.5283
FIGO stage	0.814	(0.312–2.123)	0.6734	0.729	(0.193–2.752)	0.6411
CA125 ≥ 385 U/ml	1.000	(0.999–1.000)	0.6893	1.000	(0.999–1.000)	0.5117

Hazard ratios for progress-free (PFS) and overall survival (OS), using Cox proportional hazard regression. Calculated are the 95% confidence interval (CI) of the hazard ratio and p-value in uni- and multivariable analyses for the predictive variables CTC status pre-surgery, age, R-status (reference: R0), FIGO stage (reference: FIGO IIIC), and CA125 concentrations pre-operatively.

Persistent CTC positivity (≥2 CTCs pre- and post-operative) was correlated with worse PFS (*p* = 0.0071; Supplementary Fig. 2C) in comparison to persistent CTC negativity, a decrease in CTCs post-operatively, or an increase in CTCs post-operatively. No other associations were found between CTC dynamics and survival independent of the CTC threshold (Supplementary Fig. 2).

### Post-operative CTCs and R-status combined are prognostic for worse OS

**Fig. 2:**
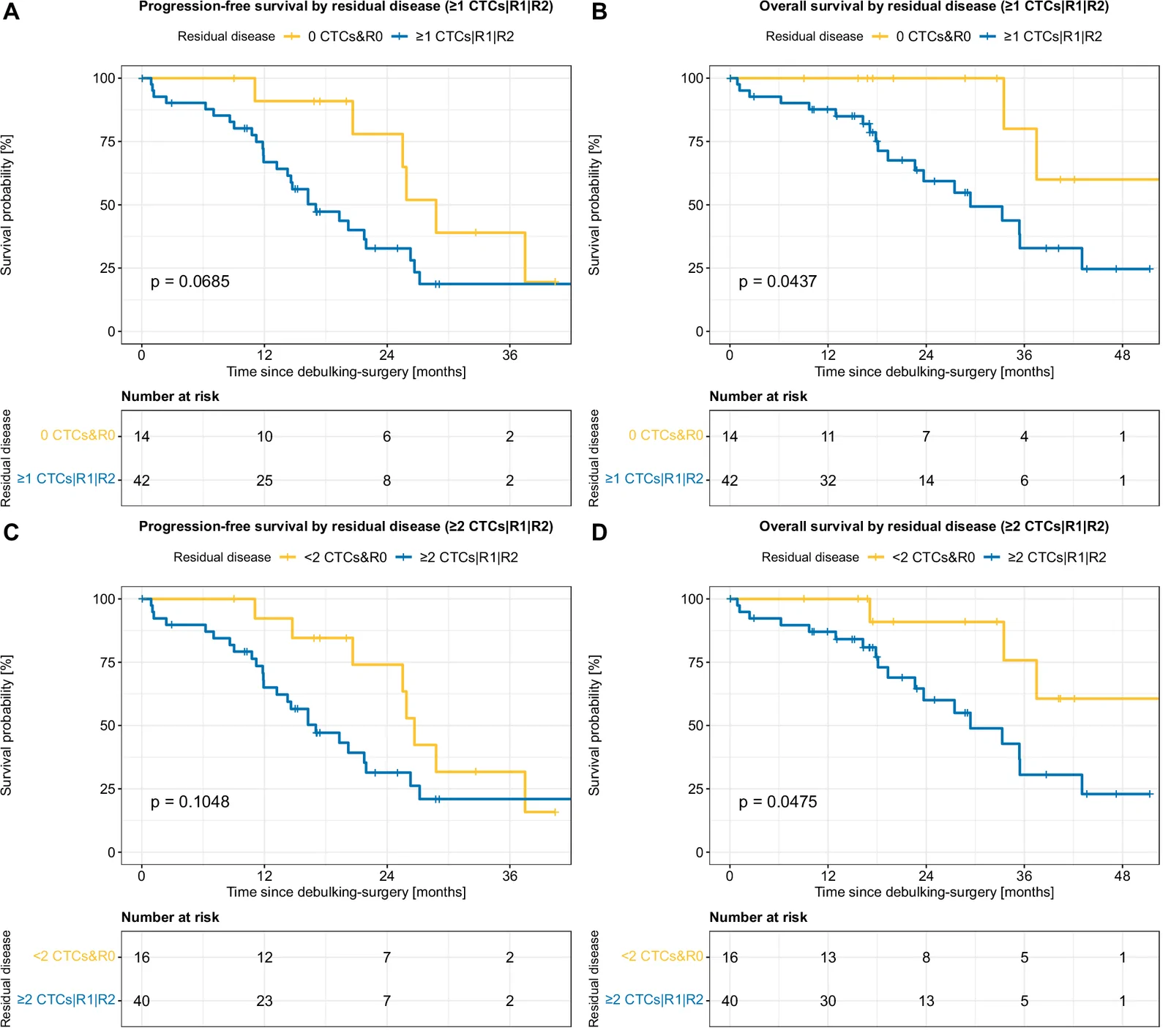
Progression-free and overall survival of HGSOC patients stratified based on presence of CTC-augmented residual disease defined by the detection of ≥1 post-operative CTCs (**A**, **B**) in combination with R1 | R2 status, and ≥2 post-operative CTCs in combination with R1 | R2 status (**C**, **D**).

A composite indicator of residual disease was defined as the presence of detectable CTCs post-operatively (≥1 and ≥2) and/or suboptimal cytoreductive surgery (R1 | R2). CTC-augmented residual disease was detected in 42 or 40 patients based on having ≥1 or ≥2 CTCs, respectively, whereas 14 or 16 patients were diagnosed with R0 and 0 or <2 CTCs post-operatively, respectively (Table 1). Although median PFS was not significantly different for patients without or with CTC-augmented residual disease (Fig. 2A, C), the median OS was significantly longer for patients without CTC-augmented residual disease (median not reached) compared to with (29.4 months, Fig. 2B, D). Moreover, the combination of R-status and ≥2 post-operative CTCs resulted in the highest concordance of 0.63 (std = 0.03) for OS in the Cox proportional hazard model (HR: 2.978, 95% CI: 1.000–8.966) compared with 0.60 (std = 0.03) for R-status and 0.56 (std = 0.04) for ≥2 post-operative CTCs alone. No association was found between PFS or OS and the number of detectable post-operative CTCs alone (Supplementary Fig. 3).

### Tumor growth patterns and CTC spread

To investigate whether specific tumor growth patterns correlate to the shedding of CTCs, all pathologically confirmed anatomical locations (*n* = 157, excluding skin) and lymph nodes with cancer that were removed during surgery (*n* = 26) were grouped by their tumor-draining veins and oncology-relevant compartments (Supplementary Table 1). Statistical significance was assessed for the presence of CTCs using both CTC cut-offs and the fraction of affected anatomical locations per location group as surrogate for tumor load per patient.

Motivated by our earlier findings of considerable local release of cells by colorectal tumors in the mesenteric vein [38] and by prostate tumors in the prostatic plexus [39], which significantly contrasted with the few cells detected in autologous peripheral blood in both cancer entities, we investigated whether the liver filters CTCs from tumors draining into the portal vein versus the inferior vena cava. However, no significant association was found based on the number of tumors draining into either vein and the presence or number of CTCs detected in peripheral blood. Grouping the tumor locations into oncology-relevant compartments, including abdominal wall, bowel, lower abdomen, lymph nodes, mesentery, pelvis, peritoneum, retroperitoneum, and upper abdomen, the detection of CTCs was significantly correlated with tumor growth in the surgically removed lymph nodes only (*r* = 0.4104, adj.p = 0.0151**;** Supplementary Table 2). Similarly, grouping the tumor locations for the affected organ’s type of tissue, including adipose, connective, epithelial, lymphatic, mesothelial, muscular, subcutaneous, and vascular tissue, only lymphatic tissue was associated with detectable CTCs (*r* = 0.4104, adj.p = 0.0134; Supplementary Table 3). From all surgically removed lymph nodes, those from the external iliac, paraaortic/caval, pelvic, common iliac, extra-abdominal, and upper abdominal nodal basins were all significantly correlated with the detection of CTCs (adj.p < 0.05; Supplementary Table 4). None of the peritoneal fluid dynamics zones were correlated with detectable CTCs in our cohort (Supplementary Table 5).

## Discussion

Identifying ovarian cancer patients at risk of incomplete tumor removal or nodal metastases before surgery could improve their clinical management. Tumor-targeted fluorescence-guided surgery and other emerging intraoperative imaging modalities have demonstrated promise in improving detection of incomplete macroscopic cytoreduction, yet remain limited in identifying microscopic or deeply invasive disease and are not routinely incorporated into current surgical guidelines [40]. Pre-operative assessment of CTCs may provide complementary predictive value by reflecting systemic tumor burden and metastatic potential beyond what is visible through pre- and intraoperative imaging. CTC analysis could enhance individualized surgical planning and identify patients at high-risk of incomplete cytoreduction and guide the use of extended interventions, including intraoperative imaging. We show that presence of ≥2 CTCs/7.5 ml of blood before debulking surgery was independently associated with higher rates of suboptimal cytoreduction, validating earlier results by Obermayr et al. [41]., shorter PFS and OS, tumor involvement in suspicious lymph nodes removed during debulking surgery, and platinum resistance. The latter finding contrasts with earlier studies; however, these studies had limited sample sizes and investigated heterogeneous groups of patients who partly received neoadjuvant chemotherapy [25, 31, 42]. Stratification of patients based on their predicted chemotherapy response may enable alternative therapeutic strategies and thereby save valuable treatment time.

Detecting pre-operative CTCs outperformed CA125 in predicting suboptimal cytoreduction and platinum response; on the other hand, the sensitivity of CTCs only in predicting R0 was lower. These results are expected and comparable with other studies that showed lower sensitivity for predicting optimal cytoreductive surgery using CA125 [15–17], possibly because of confounding factors influencing the surgery outcome such as patient fitness, surgeon skills, and available resources. In line with this, we observed that patients without detectable CTCs at any time point or showing CTC clearance post-operatively, were on average 7 years younger at diagnosis than patients without CTC clearance (mean: 61 vs. 68 years; *p* < 0.05; Table 1). This age difference may reflect that younger patients are generally fitter, possess a more robust immune response, and can tolerate more extensive debulking surgery, in addition to the possibility of having less aggressive disease; however, these hypotheses need to be systematically tested.

Reported associations between CTC status and PFS/OS of ovarian cancer patients vary widely, reflecting differences in study design, patient cohorts, blood-sampling time points, and CTC enrichment/detection methodologies [43–46]. To date, six prospective studies have used the CellSearch System to enumerate CTCs in primary ovarian cancer (Table 4). Three found no significant correlation [31, 32, 47], and three did not assess survival [25, 42, 48]. A limitation common to all these studies, including ours, is the inability to detect CTCs that downregulated EpCAM or keratin upon epithelial-to-mesenchymal transition [49–53]. Both label-independent enrichment and the adoption of other identification markers have been shown to increase CTC detection in ovarian cancer [47, 48]. These findings underscore the presence of CTCs with mesenchymal-like phenotype and that the total CTC burden is probably underestimated. Another limitation of the applied method is the inability to capture live cells such as with other CTC enrichment methods [54, 55] and the lack of further molecular characterization of identified CTCs. Single-cell molecular analyses have shown the presence of tumor cell subpopulations that correlate with tumor aggressiveness and clinical outcomes [22, 41, 56–59] and could provide information on potential druggable targets [41, 60]. Nevertheless, although label-independent approaches may enhance detection, the standardized nature of the CellSearch System and the reproducibility of its results facilitate robust comparisons among studies and support its integration into clinical trials.

**Table 4:** CTC studies.

Reference	Study type	Time point	FIGO	Cohort	Sample size	CTC threshold	CTC positivity	Mean/median CTC	OS	PFS	Aim
Banys-Paluchowski et al. [32]	Prospective	BL (pre-chemo), after cycle 3 and 6	I–IV	Primary ovarian, fallopian tube and peritoneal cancer	23	≥1 CTCs/7.5 ml	17%	NA	*p* = 0.192	*p* = 0.085	Evaluate CTC dynamic and clinical relevance
^a^ Obermayr et al. [46]	Prospective	BL (at diagnosis), 6 months after chemotherapy	II–IV	Primary ovarian cancer	139	NA	3.6%	NA	NA	NA	Assess tumor load/MRD
Abreu et al. [45]	Prospective	BL (pre-treatment), after cycle 3 and 6	I–IV	Primary HGSOC	25	≥1 CTCs/7.5 ml	21%	Range 1–8	*p* > 0.05	*p* > 0.05	CTC characterization
Liu et al. [31]	Prospective	BL (pre-chemo), after cycle 3 and 6	III–IV	Primary ovarian, fallopian tube and peritoneal cancer	30	≥2 CTCs/7.5 ml	82%	Range 0–6, median 2	*p* = 0.40	*p* = 0.88	Prediction of clinical outcomes
Lou et al. [25]	Prospective	BL ( = at diagnosis)	I–IV	Primary LGSOC and HGSOC	23	≥1 CTCs/7.5 ml	21.70%	NA	NA	NA	Evaluate CTCs as predictive marker for platinum resistance
Lou et al. [47]	Prospective	BL ( = pre-treatment	I–IV	Primary ovarian cancer	49	≥1 CTCs/7.5 ml	17.2%	1-21, median 2	NA	NA	CTCs as diagnostic marker

Prospective and clinical studies analyzing quantitative CTC data obtained using the CellSearch® System in primary ovarian cancer.

*OS* overall survival, *PFS* Progression-free survival, *NA* not available, *EOC* epithelial ovarian cancer, *HGSOC* high-grade serous ovarian cancer, *LGSOC* Low-grade serous ovarian cancer, *BL* baseline a) applied CellSearch® System after density-gradient centrifugation of 20 ml blood and aborted CellSearch as CTC enrichment method mid-study, due to low number of CTC-positive samples.

HGSOC mainly spreads within the pelvic cavity through the peritoneal fluid, whereas hematogenous dissemination is rare and more likely to occur once the tumor invades lymphatic or blood vessels [32, 61–67]. In line with this, we observed that patients with tumor growth in suspicious lymph nodes were more likely to have detectable CTCs in peripheral blood. However, the detected metastases draining into the portal vein were not associated with a decreased detection of peripheral CTCs, as we previously described in colorectal cancer [38]. It should be noted that our analysis of hematogenous routes of tumor spread in HGSOC should be considered as hypothesis generating only, because the tumor load per affected organ was not quantitatively and systematically recorded. We speculate that ovarian cancer metastasizing through the peritoneal fluid and growing onto other organs (from the outside-in), may lead to differences in the extent of intravasation compared to primary tumors infiltrating vessels from within the organ of origin (from the inside-out). As such, CTC detection in blood may serve as a surrogate marker for nodal involvement and reflect higher tumor burden, invasiveness, or tumor cell plasticity rather than indicating a specific metastatic pathway. CTCs indirectly indicate aggressive invasion and growth patterns that may be responsible for the lack of complete surgical resectability.

Post-operative CTC levels were not directly associated with therapy outcome, possibly because the dynamics of CTC release from different tumor populations were altered as a response to surgery-induced wound-healing, temporarily increased innate immune activity, and circulating phagocytes [68–70]. However, post-operative CTCs in combination with R-status, here defined as CTC-augmented residual disease, were significantly associated with overall survival. Therefore, early post-operative CTC counts alone provided limited prognostic value, and further research is required to establish whether CTC-based minimal residual disease (MRD) can be detected several days or even weeks later.

Our results could help in guiding patient stratification and predict the benefit of alternative treatments, such as neoadjuvant chemotherapy (NACT) with interval debulking surgery (IDS) or hyperthermic intraperitoneal chemotherapy. In unstratified ovarian cancer patients, NACT/IDS does not result in superior PFS/OS compared to primary debulking surgery followed by chemotherapy [71–74]. In the recently presented TRUST Study, comparing primary debulking surgery with NACT/IDS in advanced ovarian cancer patients with expected complete resectable tumors, it was shown that only patients with complete resectable tumors had a survival benefit from primary surgery [74]. On the other hand, significant survival improvements can be achieved if NACT is followed by hyperthermic intraperitoneal chemotherapy in combination with IDS as compared to IDS alone [75–77]. In routine clinical care, it can take several weeks from referral to the diagnostic procedure taking place, providing a window, particularly at the time of diagnostic laparoscopy and prior debulking surgery, during which blood samples could be obtained for CTC enrichment and analysis within two days. Although our results are promising, inclusion of CTC enumeration in future clinical trials will be required to provide further evidence before CTC-based intervention trials can be initiated.

In conclusion, pre-operative CTC counts of ≥2 in primary, advanced-stage HGSOC patients were significantly and independently correlated with worse PFS and OS, a higher risk of incomplete resection, tumor growth in suspicious lymph nodes, and resistance to platinum therapy. Although further research is required to validate these results, pre-operative CTCs may be used to identify high-risk patients who might benefit from alternative treatments, and thereby reduce surgical morbidity, optimize resource allocation, and strengthen patient counseling.

## Supplementary information

SUPPLEMENTAL MATERIAL

## Data Availability

All data generated or analyzed during this study are included in this published article and its supplementary information files.
