# Supplementary material for: Pre-operative circulating tumor cells predict worse treatment outcome in patients with high-grade serous ovarian cancer
Source: Oncogene. 2026 Apr 4;45(18):1688–99. doi: 10.1038/s41388-026-03747-3 (PMC13558050; doi:10.1038/s41388-026-03747-3)
Supplement: Supplementary file 1 [file 41388_2026_3747_MOESM1_ESM.docx]

**Supplementary Figure 1**: Association between residual tumor and (A) progression-free survival and (B) overall survival in patients diagnosed with advanced high-grade serous ovarian cancer. R0=optimal debulking with complete macroscopic tumor resection, R1=residual tumor of ≤1 cm, and R2=residual tumor >1 cm. In multivariable analyses, patients diagnosed with R2 had significantly increased risk of progression in comparison to patients with R0 (HR=3.40, 95%CI: 1.29-8.98, p=0.0134), whereas no increased risk could be detected for the patients diagnosed with R1. No significant association was found between FIGO stage and residual tumor status (p=0.1768), suggesting that the observed impact of residual tumor on PFS is not confounded by FIGO stage. Patients diagnosed with R2 in comparison to R0 had a higher risk of shorter OS (HR=4.03, 95%CI: 1.19-13.68, p=0.0255). Due to the limited sample size, no statistically significant correlation was found between residual tumor status and OS. Residual disease is a known predictor of PFS, which we confirm. Overall, these results show that the cohort used for analysis in this study is a representative HGSOC cohort.

**Supplementary Figure 2**: Association between dynamics of circulating tumor cells (CTCs) from pre- to post-operative time point and (A) progression-free survival and (B) overall survival based on the presence of ≥1 CTCs and (C) progression-free survival and (D) overall survival based on the presence of ≥2 CTCs in patients diagnosed with advanced high-grade serous ovarian cancer. Persistent negative=CTCs below threshold pre- and post-operatively; Decrease=CTCs above threshold pre- and below threshold post-operatively; Increase=CTCs below threshold pre- and above threshold post-operatively; Persistent positive=CTCs above threshold at both time points.

**Supplementary Figure 3**: Association between post-operative circulating tumor cells (CTCs) and (A) progression-free survival and (B) overall survival based on the presence of ≥1 CTCs and (C) progression-free survival and (D) overall survival based on the presence of ≥2 CTCs in patients diagnosed with advanced high-grade serous ovarian cancer. The detection of ≥1 CTCs post-operatively was not correlated with PFS (median: 21.7 and 16.3 months, p=0.5499) and OS (median: 35.3 and 35.4 months, p=0.2761), the presence of ≥2 CTCs post-operatively was not correlated with PFS (median: 21.6 and 14.3 months, p=0.1903) and OS (median: 35.4 and 42.9 months, p=0.6995).

**Supplementary Table 1**: Pathological confirmed anatomical locations (n=157, excluding skin) and suspect lymph nodes that were removed based on the surgeon’s decision (n=26) from 56 high-grade serous ovarian cancer patients grouped by their tumor draining veins and oncology relevant compartments.

| **Anatomical locations with pathologically confirmed metastasis** | **Venous drainage** | **Direct venous outflow** | **IVC/Portal drainage** | **Oncology relevant compartment** | **Oncology relevant nodal basins** | **Tissue type** | **Peritoneal Fluid Dynamics Zones** |
| --- | --- | --- | --- | --- | --- | --- | --- |
| Abdominal wall | Thoracoepigastric veins → subclavian/external iliac veins | Thoracoepigastric veins | Inferior vena cava | Abdominal wall |  | Muscular | Anterior abdominal peritoneum |
| Anterior abdominal wall | Thoracoepigastric veins → subclavian veins (above umbilicus), external iliac veins (below umbilicus) | Thoracoepigastric veins | Inferior vena cava | Abdominal wall |  | Muscular | Anterior abdominal peritoneum |
| Anterior abdominal wall peritoneum | Thoracoepigastric veins → subclavian/external iliac veins | Thoracoepigastric veins | Inferior vena cava | Abdominal wall |  | Mesothelial | Anterior abdominal peritoneum |
| Left lower abdominal peritoneum | Inferior mesenteric vein → portal vein | Inferior mesenteric vein | Portal | Lower abdomen |  | Mesothelial | Anterior abdominal peritoneum |
| Left lower abdominal puncture site | Inferior epigastric vein → external iliac vein | Inferior epigastric vein | Inferior vena cava | Abdominal wall |  | Subcutaneous | Anterior abdominal peritoneum |
| Left lower abdominal subcutaneous trocar insertion | Inferior epigastric vein → external iliac vein | Inferior epigastric vein | Inferior vena cava | Abdominal wall |  | Subcutaneous | Anterior abdominal peritoneum |
| Left lower abdominal trocar insertion site | Inferior epigastric vein → external iliac vein | Inferior epigastric vein | Inferior vena cava | Abdominal wall |  | Subcutaneous | Anterior abdominal peritoneum |
| Left pelvic wall | Internal iliac vein | Internal iliac vein | Inferior vena cava | Pelvis |  | Muscular | Anterior abdominal peritoneum |
| Left trocar insertion site | Inferior epigastric vein → external iliac vein | Inferior epigastric vein | Inferior vena cava | Abdominal wall |  | Subcutaneous | Anterior abdominal peritoneum |
| Left umbilicus | Paraumbilical veins → portal vein | Paraumbilical veins | Portal | Abdominal wall |  | Subcutaneous | Anterior abdominal peritoneum |
| Midline abdominal wall | Superior and inferior epigastric veins → internal thoracic vein / external iliac vein | Inferior epigastric vein | Inferior vena cava | Abdominal wall |  | Muscular | Anterior abdominal peritoneum |
| Parietal peritoneum | Portal venous system (mesenteric veins) and systemic veins | Portal vein | Portal | Peritoneum |  | Mesothelial | Anterior abdominal peritoneum |
| Right abdominal wall peritoneum | Inferior epigastric vein → external iliac vein | Inferior epigastric vein | Inferior vena cava | Abdominal wall |  | Mesothelial | Anterior abdominal peritoneum |
| Right lower abdominal fascia | Inferior epigastric vein → external iliac vein | Inferior epigastric vein | Inferior vena cava | Abdominal wall |  | Connective | Anterior abdominal peritoneum |
| Right lower abdominal peritoneum | Inferior mesenteric vein → portal vein | Inferior mesenteric vein | Portal | Lower abdomen |  | Mesothelial | Anterior abdominal peritoneum |
| Right lower abdominal puncture site | Inferior epigastric vein → external iliac vein | Inferior epigastric vein | Inferior vena cava | Abdominal wall |  | Subcutaneous | Anterior abdominal peritoneum |
| Right lower abdominal trocar insertion site | Inferior epigastric vein → external iliac vein | Inferior epigastric vein | Inferior vena cava | Abdominal wall |  | Subcutaneous | Anterior abdominal peritoneum |
| Right pelvic wall | Internal iliac vein | Internal iliac vein | Inferior vena cava | Pelvis |  | Muscular | Anterior abdominal peritoneum |
| Right trocar insertion site | Inferior epigastric vein → external iliac vein | Inferior epigastric vein | Inferior vena cava | Abdominal wall |  | Subcutaneous | Anterior abdominal peritoneum |
| Scar peritoneum | Portal venous system (mesenteric veins) and systemic veins | Portal vein | Portal | Abdominal wall |  | Mesothelial | Anterior abdominal peritoneum |
| Subumbilical peritoneum | Inferior epigastric vein → external iliac vein / paraumbilical veins → portal vein | Inferior epigastric vein | Inferior vena cava | Abdominal wall |  | Mesothelial | Anterior abdominal peritoneum |
| Umbilical puncture site | Inferior epigastric vein → external iliac vein | Inferior epigastric vein | Inferior vena cava | Abdominal wall |  | Subcutaneous | Anterior abdominal peritoneum |
| Umbilical trocar insertion site | Inferior epigastric vein → external iliac vein | Inferior epigastric vein | Inferior vena cava | Abdominal wall |  | Subcutaneous | Anterior abdominal peritoneum |
| Umbilicus | Paraumbilical veins → portal vein | Paraumbilical veins | Portal | Abdominal wall |  | Subcutaneous | Anterior abdominal peritoneum |
| Ileocecal mesentery | Ileocolic vein → superior mesenteric vein → portal vein | Ileocolic vein | Portal | Mesentery |  | Connective | Inframesocolic compartment |
| Ileum with mesentery | Ileal veins → superior mesenteric vein → portal vein | Ileal veins | Portal | Mesentery |  |  | Inframesocolic compartment |
| Jejunal deposit | Jejunal veins → superior mesenteric vein → portal vein | Jejunal veins | Portal | Upper abdomen |  | Epithelial | Inframesocolic compartment |
| Ligament of Treitz | Superior mesenteric vein → portal vein | Superior mesenteric vein | Portal | Upper abdomen |  | Connective | Inframesocolic compartment |
| Mesentery | Superior mesenteric vein / inferior mesenteric vein → portal vein | Superior mesenteric vein | Portal | Mesentery |  | Connective | Inframesocolic compartment |
| Meso of small intestine | Superior mesenteric vein → portal vein | Superior mesenteric vein | Portal | Mesentery |  | Connective | Inframesocolic compartment |
| Mesocolon | Colic veins → superior/inferior mesenteric veins → portal vein | Colic veins | Portal | Mesentery |  | Connective | Inframesocolic compartment |
| Mesoileum | Ileal veins → superior mesenteric vein → portal vein | Ileal veins | Portal | Mesentery |  | Connective | Inframesocolic compartment |
| Mesojejunum | Jejunal veins → superior mesenteric vein → portal vein | Jejunal veins | Portal | Mesentery |  | Connective | Inframesocolic compartment |
| Mesosigmoid peritoneum | Inferior mesenteric vein → portal vein | Inferior mesenteric vein | Portal | Mesentery |  | Mesothelial | Inframesocolic compartment |
| Root of mesentery deposit | Mesenteric veins → portal vein | Mesenteric veins | Portal | Mesentery |  | Connective | Inframesocolic compartment |
| Small intestine mesentery | Mesenteric veins → portal vein | Mesenteric veins | Portal | Mesentery |  | Connective | Inframesocolic compartment |
| Tumor deposit on mesosigmoid | Inferior mesenteric vein → portal vein | Inferior mesenteric vein | Portal | Mesentery |  | Connective | Inframesocolic compartment |
| Left paracolic gutter | Inferior mesenteric vein → portal vein | Inferior mesenteric vein | Portal | Peritoneum |  |  | Left paracolic gutter |
| Left paracolic gutter peritoneum | Inferior mesenteric vein → portal vein | Inferior mesenteric vein | Portal | Peritoneum |  | Mesothelial | Left paracolic gutter |
| Mesentery of descending colon | Inferior mesenteric vein → portal vein | Inferior mesenteric vein | Portal | Mesentery |  | Connective | Left paracolic gutter |
| Dorsal spleen area | Splenic vein → portal vein | Splenic vein | Portal | Upper abdomen |  | Epithelial | Left subphrenic space |
| Left diaphragmatic dome | Inferior phrenic vein → IVC | Inferior phrenic vein | Inferior vena cava | Upper abdomen |  | Muscular | Left subphrenic space |
| Left diaphragmatic dome peritoneum | Inferior phrenic vein → IVC | Inferior phrenic vein | Inferior vena cava | Upper abdomen |  | Mesothelial | Left subphrenic space |
| Left diaphragmatic peritoneum | Inferior phrenic vein → IVC | Inferior phrenic vein | Inferior vena cava | Upper abdomen |  | Mesothelial | Left subphrenic space |
| Spleen | Splenic vein → portal vein | Splenic vein | Portal | Upper abdomen |  | Epithelial | Left subphrenic space |
| Splenic hilum | Splenic vein → portal vein | Splenic vein | Portal | Upper abdomen |  | Connective | Left subphrenic space |
| Lesser omentum | Left gastric vein → portal vein | Gastric veins | Portal | Upper abdomen |  | Adipose | Lesser sac (omental bursa) |
| Omental bursa | Left gastric vein / splenic vein → portal vein | Gastric veins | Portal | Upper abdomen |  | Mesothelial | Lesser sac (omental bursa) |
| Posterior stomach wall deposit | Short gastric veins → splenic vein → portal vein | Gastric veins | Portal | Upper abdomen |  | Epithelial | Lesser sac (omental bursa) |
| Retrogastric deposit | Left gastric vein → portal vein | Gastric veins | Portal | Upper abdomen |  | Epithelial | Lesser sac (omental bursa) |
| Appendix | Appendicular vein → ileocolic vein → superior mesenteric vein → portal vein | Appendicular vein | Portal | Bowel |  | Epithelial |  |
| Ascending colon | Superior mesenteric vein → portal vein | Superior mesenteric vein | Portal | Bowel |  | Epithelial |  |
| Bladder | Vesical venous plexus → internal iliac vein | Uterine/Vaginal/Vesical venous plexus | Inferior vena cava | Pelvis |  | Epithelial |  |
| Cecal pole | Ileocolic vein → superior mesenteric vein → portal vein | Ileocolic vein | Portal | Bowel |  | Epithelial |  |
| Cecum | Ileocolic vein → superior mesenteric vein → portal vein | Ileocolic vein | Portal | Bowel |  | Epithelial |  |
| Cervix | Uterine venous plexus → internal iliac vein | Uterine/Vaginal/Vesical venous plexus | Inferior vena cava | Pelvis |  | Epithelial |  |
| Collar pouch | Internal iliac vein | Internal iliac vein | Inferior vena cava | Pelvis |  | Mesothelial |  |
| Colon | Colic veins (superior + inferior mesenteric veins) → portal vein | Colic veins | Portal | Bowel |  | Epithelial |  |
| Descending colon | Inferior mesenteric vein → portal vein | Inferior mesenteric vein | Portal | Bowel |  | Epithelial |  |
| Diaphragm | Inferior phrenic veins → IVC / superior phrenic → azygos | Inferior phrenic vein | Inferior vena cava | Upper abdomen |  | Muscular |  |
| Diaphragmatic peritoneum | Inferior phrenic veins → IVC | Inferior phrenic vein | Inferior vena cava | Upper abdomen |  | Mesothelial |  |
| Distal pancreas | Splenic vein → portal vein | Splenic vein | Portal | Upper abdomen |  | Epithelial |  |
| Duodenum | Superior pancreaticoduodenal vein → portal vein | Portal vein | Portal | Upper abdomen |  | Epithelial |  |
| Epiploic appendix | Colic veins → superior mesenteric vein → portal vein | Colic veins | Portal | Bowel |  | Adipose |  |
| Fimbrial funnel | Ovarian vein → left renal vein (left) / IVC (right) | Ovarian vein | Inferior vena cava | Pelvis |  | Epithelial |  |
| Gallbladder | Cystic vein → portal vein | Cystic vein | Portal | Upper abdomen |  | Epithelial |  |
| Gerota’s fascia | Renal veins → IVC | Renal vein | Inferior vena cava | Retroperitoneum |  | Connective |  |
| Greater curvature of stomach | Left + right gastroepiploic veins → splenic / SMV → portal vein | Gastroepiploic veins | Portal | Upper abdomen |  | Epithelial |  |
| Greater omentum | Right and left gastroepiploic veins → superior mesenteric / splenic veins → portal vein | Gastroepiploic veins | Portal | Upper abdomen |  | Adipose |  |
| Hepatoduodenal ligament | Portal vein (formed by superior mesenteric vein + splenic vein) | Portal vein | Portal | Upper abdomen |  | Connective |  |
| Hernia sac | Thoracoepigastric veins → subclavian/external iliac veins | Thoracoepigastric veins | Inferior vena cava | Abdominal wall |  | Connective |  |
| Ileocecal pole | Ileocolic vein → superior mesenteric vein → portal vein | Ileocolic vein | Portal | Bowel |  | Epithelial |  |
| Ileocecal region | Ileocolic vein → superior mesenteric vein → portal vein | Ileocolic vein | Portal | Bowel |  | Epithelial |  |
| Ileum | Ileal veins → superior mesenteric vein → portal vein | Ileal veins | Portal | Bowel |  | Epithelial |  |
| Inferior epigastric artery | Inferior epigastric vein → external iliac vein | Inferior epigastric vein | Inferior vena cava |  |  | Vascular |  |
| Inferior mesenteric artery | Inferior mesenteric vein → portal vein | Inferior mesenteric vein | Portal |  |  | Vascular |  |
| Intestinal serosa | Mesenteric veins → portal vein | Mesenteric veins | Portal | Peritoneum |  | Mesothelial |  |
| Large intestine | Superior & inferior mesenteric veins → portal vein | Superior mesenteric vein | Portal | Bowel |  | Epithelial |  |
| Left adnexa | Ovarian vein → IVC (right) / left renal vein (left) | Ovarian vein | Inferior vena cava | Pelvis |  | Epithelial |  |
| Left colic flexure | Inferior mesenteric vein → portal vein | Inferior mesenteric vein | Portal | Bowel |  | Epithelial |  |
| Left colonic fossa | Inferior mesenteric vein → portal vein | Inferior mesenteric vein | Portal | Bowel |  | Connective |  |
| Left common iliac artery | Common iliac vein → inferior vena cava | Common iliac vein | Inferior vena cava | Retroperitoneum |  | Vascular |  |
| Left external iliac artery | External iliac vein → common iliac vein → inferior vena cavaExternal iliac vein | Common iliac vein | Inferior vena cava | Retroperitoneum |  | Vascular |  |
| Left fallopian tube | Ovarian vein → IVC (right) / left renal vein (left) | Ovarian vein | Inferior vena cava | Pelvis |  | Epithelial |  |
| Left fimbrial funnel | Left ovarian vein → left renal vein | Ovarian vein | Inferior vena cava | Pelvis |  | Epithelial |  |
| Left hemicolectomy | Inferior mesenteric vein → portal vein | Inferior mesenteric vein | Portal | Bowel |  |  |  |
| Left lower abdomen | Inferior epigastric vein → external iliac vein | Inferior epigastric vein | Inferior vena cava | Lower abdomen |  |  |  |
| Left ovarian vascular bundle | Left ovarian vein → left renal vein | Ovarian vein | Inferior vena cava | Pelvis |  | Vascular |  |
| Ligamentum teres hepatis and falciform ligament | Paraumbilical veins → portal vein | Paraumbilical veins | Portal | Upper abdomen |  | Connective |  |
| Liver | Hepatic veins → inferior vena cava | Hepatic veins | Inferior vena cava | Upper abdomen |  | Epithelial |  |
| Liver capsule | Hepatic veins → inferior vena cava | Hepatic veins | Inferior vena cava | Upper abdomen |  | Connective |  |
| Lymph node, caval |  |  |  | Lymph nodes | Paraaortic / Caval | Lymphatic |  |
| Lymph node, common iliac |  |  |  | Lymph nodes | Common iliac | Lymphatic |  |
| Lymph node, deep inguinal |  |  |  | Lymph nodes | Inguinal | Lymphatic |  |
| Lymph node, inferior mesenteric |  |  |  | Lymph nodes | Upper abdominal | Lymphatic |  |
| Lymph node, interaortocaval |  |  |  | Lymph nodes | Paraaortic / Caval | Lymphatic |  |
| Lymph node, internal iliac |  |  |  | Lymph nodes | Internal iliac | Lymphatic |  |
| Lymph node, left axillary |  |  |  | Lymph nodes | Extra-abdominal | Lymphatic |  |
| Lymph node, left common iliac |  |  |  | Lymph nodes | Common iliac | Lymphatic |  |
| Lymph node, left external iliac |  |  |  | Lymph nodes | External iliac | Lymphatic |  |
| Lymph node, left inguinal |  |  |  | Lymph nodes | Inguinal | Lymphatic |  |
| Lymph node, left internal iliac |  |  |  | Lymph nodes | Internal iliac | Lymphatic |  |
| Lymph node, left paraaortic |  |  |  | Lymph nodes | Paraaortic / Caval | Lymphatic |  |
| Lymph node, left pelvic |  |  |  | Lymph nodes | Pelvic | Lymphatic |  |
| Lymph node, pancreatic |  |  |  | Lymph nodes | Upper abdominal | Lymphatic |  |
| Lymph node, paraaortic |  |  |  | Lymph nodes | Paraaortic / Caval | Lymphatic |  |
| Lymph node, paracaval |  |  |  | Lymph nodes | Paraaortic / Caval | Lymphatic |  |
| Lymph node, pararectal |  |  |  | Lymph nodes | Pelvic | Lymphatic |  |
| Lymph node, peripancreatic |  |  |  | Lymph nodes | Upper abdominal | Lymphatic |  |
| Lymph node, presacral |  |  |  | Lymph nodes | Pelvic | Lymphatic |  |
| Lymph node, right common iliac |  |  |  | Lymph nodes | Common iliac | Lymphatic |  |
| Lymph node, right external iliac |  |  |  | Lymph nodes | External iliac | Lymphatic |  |
| Lymph node, right internal iliac |  |  |  | Lymph nodes | Internal iliac | Lymphatic |  |
| Lymph node, right obturator |  |  |  | Lymph nodes | Pelvic | Lymphatic |  |
| Lymph node, right paraaortic |  |  |  | Lymph nodes | Paraaortic / Caval | Lymphatic |  |
| Lymph node, right pararectal |  |  |  | Lymph nodes | Pelvic | Lymphatic |  |
| Lymph node, right pelvic |  |  |  | Lymph nodes | Pelvic | Lymphatic |  |
| Mesocolon at left colic flexure | Inferior mesenteric vein → portal vein | Inferior mesenteric vein | Portal | Mesentery |  | Connective |  |
| Omentum | Gastroepiploic veins → splenic/superior mesenteric veins → portal vein | Gastroepiploic veins | Portal | Upper abdomen |  | Adipose |  |
| Peristomal skin |  |  |  |  |  | Epithelial |  |
| Peritoneum | Mesenteric veins → portal vein | Mesenteric veins | Portal | Peritoneum |  | Mesothelial |  |
| Proximal ureter | Renal veins → IVC | Renal vein | Inferior vena cava | Retroperitoneum |  | Epithelial |  |
| Right adnexa | Right ovarian vein → IVC | Ovarian vein | Inferior vena cava | Pelvis |  | Epithelial |  |
| Right diaphragm | Right inferior phrenic vein → IVC | Inferior phrenic vein | Inferior vena cava | Upper abdomen |  | Muscular |  |
| Right fallopian tube | Ovarian vein → IVC (right) / left renal vein (left) | Ovarian vein | Inferior vena cava | Pelvis |  | Epithelial |  |
| Right Gerota’s fascia | Renal veins → IVC | Renal vein | Inferior vena cava | Retroperitoneum |  | Connective |  |
| Right hemicolon | Ileocolic + right colic + middle colic veins → superior mesenteric vein → portal vein | Ileocolic vein | Portal | Bowel |  | Epithelial |  |
| Right hemicolon with terminal ileum | Ileocolic + right colic + middle colic veins → superior mesenteric vein → portal vein | Ileocolic vein | Portal | Bowel |  | Epithelial |  |
| Right lower abdomen | Inferior epigastric vein → external iliac vein | Inferior epigastric vein | Inferior vena cava | Lower abdomen |  |  |  |
| Right ovarian vascular bundle | Right ovarian vein → IVC | Ovarian vein | Inferior vena cava | Pelvis |  | Vascular |  |
| Right round ligament | Paraumbilical veins → portal vein | Paraumbilical veins | Portal | Pelvis |  | Connective |  |
| Round ligament | Paraumbilical veins → portal vein | Paraumbilical veins | Portal | Pelvis |  | Connective |  |
| Sigmoid colon | Inferior mesenteric vein → portal vein | Inferior mesenteric vein | Portal | Bowel |  | Epithelial |  |
| Sigmoid peritoneal deposit | Inferior mesenteric vein → portal vein | Inferior mesenteric vein | Portal | Peritoneum |  | Mesothelial |  |
| Small intestine | Superior mesenteric vein → portal vein | Superior mesenteric vein | Portal | Bowel |  | Epithelial |  |
| Spleen margin area | Splenic vein → portal vein | Splenic vein | Portal | Upper abdomen |  | Epithelial |  |
| Stomach | Gastric veins (left, right) + gastroepiploic veins + short gastric veins → portal vein | Gastric veins | Portal | Upper abdomen |  | Epithelial |  |
| Stomach antrum/corpus/fundus | Left/right gastric + short gastric + gastroepiploic veins → portal vein | Gastric veins | Portal | Upper abdomen |  | Epithelial |  |
| Terminal ileum | Ileal veins → superior mesenteric vein → portal vein | Ileal veins | Portal | Bowel |  | Epithelial |  |
| Transverse colon | Middle colic vein → superior mesenteric vein → portal vein | Middle colic vein | Portal | Bowel |  | Epithelial |  |
| Transverse mesocolon | Middle colic vein → superior mesenteric vein → portal vein | Middle colic vein | Portal | Mesentery |  | Connective |  |
| Umbilical hernia peritoneum | Paraumbilical veins → portal vein | Paraumbilical veins | Portal | Abdominal wall |  | Mesothelial |  |
| Umbilical hernia sac | Paraumbilical veins → portal vein | Paraumbilical veins | Portal | Abdominal wall |  | Connective |  |
| Uterus | Uterine veins → internal iliac vein | Uterine/Vaginal/Vesical venous plexus | Inferior vena cava | Pelvis |  | Epithelial |  |
| Vermiform appendix | Appendicular vein → ileocolic vein → superior mesenteric vein → portal vein | Appendicular vein | Portal | Bowel |  | Epithelial |  |
| Vulva cranial right 11 o’clock | Pudendal vein → internal iliac vein | Internal iliac vein | Inferior vena cava | Pelvis |  | Epithelial |  |
| Vulva left 2 o’clock | Pudendal vein → internal iliac vein | Internal iliac vein | Inferior vena cava | Pelvis |  | Epithelial |  |
| Vulvar introitus left 3 o’clock | Pudendal vein → internal iliac vein | Internal iliac vein | Inferior vena cava | Pelvis |  | Epithelial |  |
| Anterior rectum | Superior rectal vein → inferior mesenteric vein → portal vein | Superior rectal vein | Portal | Bowel |  | Epithelial | Pelvic cavity (Douglas pouch) |
| Bladder peritoneum | Vesical venous plexus → internal iliac vein | Uterine/Vaginal/Vesical venous plexus | Inferior vena cava | Pelvis |  | Mesothelial | Pelvic cavity (Douglas pouch) |
| Left obturator fossa | Obturator vein → internal iliac vein | Obturator vein | Inferior vena cava | Pelvis |  | Connective | Pelvic cavity (Douglas pouch) |
| Left pelvic peritoneum | Internal iliac vein | Internal iliac vein | Inferior vena cava | Pelvis |  | Mesothelial | Pelvic cavity (Douglas pouch) |
| Left pelvic wall peritoneum | Internal iliac vein | Internal iliac vein | Inferior vena cava | Pelvis |  | Mesothelial | Pelvic cavity (Douglas pouch) |
| Left pelvis | Internal iliac vein | Internal iliac vein | Inferior vena cava | Pelvis |  |  | Pelvic cavity (Douglas pouch) |
| Mesorectum | Superior rectal vein → inferior mesenteric vein → portal vein | Superior rectal vein | Portal | Mesentery |  | Connective | Pelvic cavity (Douglas pouch) |
| Obturator fossa | Obturator vein → internal iliac vein | Obturator vein | Inferior vena cava | Pelvis |  | Connective | Pelvic cavity (Douglas pouch) |
| Pararectal tumor | Superior rectal vein → inferior mesenteric vein → portal vein | Superior rectal vein | Portal | Pelvis |  | Epithelial | Pelvic cavity (Douglas pouch) |
| Pelvic peritoneum | Internal iliac vein | Internal iliac vein | Inferior vena cava | Pelvis |  | Mesothelial | Pelvic cavity (Douglas pouch) |
| Pelvic wall peritoneum | Internal iliac vein | Internal iliac vein | Inferior vena cava | Pelvis |  | Mesothelial | Pelvic cavity (Douglas pouch) |
| Posterior bladder wall | Vesical venous plexus → internal iliac vein | Uterine/Vaginal/Vesical venous plexus | Inferior vena cava | Pelvis |  | Epithelial | Pelvic cavity (Douglas pouch) |
| Pouch of Douglas peritoneum | Superior rectal vein → inferior mesenteric vein → portal vein | Superior rectal vein | Portal | Pelvis |  | Mesothelial | Pelvic cavity (Douglas pouch) |
| Rectosigmoid | Superior rectal vein → inferior mesenteric vein → portal vein | Superior rectal vein | Portal | Bowel |  | Epithelial | Pelvic cavity (Douglas pouch) |
| Rectovaginal septum | Vaginal venous plexus → internal iliac vein | Uterine/Vaginal/Vesical venous plexus | Inferior vena cava | Pelvis |  | Connective | Pelvic cavity (Douglas pouch) |
| Rectum | Superior rectal vein → inferior mesenteric vein → portal vein; middle & inferior rectal veins → internal iliac vein | Superior rectal vein | Portal | Bowel |  | Epithelial | Pelvic cavity (Douglas pouch) |
| Right obturator fossa | Obturator vein → internal iliac vein | Obturator vein | Inferior vena cava | Pelvis |  | Connective | Pelvic cavity (Douglas pouch) |
| Right pelvic peritoneum | Internal iliac vein | Internal iliac vein | Inferior vena cava | Pelvis |  | Mesothelial | Pelvic cavity (Douglas pouch) |
| Right pelvic wall peritoneum | Internal iliac vein | Internal iliac vein | Inferior vena cava | Pelvis |  | Mesothelial | Pelvic cavity (Douglas pouch) |
| Right pelvis | Internal iliac vein | Internal iliac vein | Inferior vena cava | Pelvis |  |  | Pelvic cavity (Douglas pouch) |
| Small pelvis peritoneum | Internal iliac vein | Internal iliac vein | Inferior vena cava | Pelvis |  | Mesothelial | Pelvic cavity (Douglas pouch) |
| Tangential rectum | Superior rectal vein → inferior mesenteric vein → portal vein | Superior rectal vein | Portal | Bowel |  | Epithelial | Pelvic cavity (Douglas pouch) |
| True pelvis | Internal iliac vein | Internal iliac vein | Inferior vena cava | Pelvis |  |  | Pelvic cavity (Douglas pouch) |
| Vaginal cuff | Vaginal venous plexus → internal iliac vein | Uterine/Vaginal/Vesical venous plexus | Inferior vena cava | Pelvis |  | Epithelial | Pelvic cavity (Douglas pouch) |
| Ascending colon peritoneum | Superior mesenteric vein → portal vein | Superior mesenteric vein | Portal | Peritoneum |  | Mesothelial | Right paracolic gutter |
| Cecal peritoneum | Ileocolic vein → superior mesenteric vein → portal vein | Ileocolic vein | Portal | Peritoneum |  | Mesothelial | Right paracolic gutter |
| Right colic flexure | Right colic vein → superior mesenteric vein → portal vein | Gastroepiploic veins | Portal | Bowel |  | Epithelial | Right paracolic gutter |
| Right paracolic gutter | Superior mesenteric vein → portal vein | Superior mesenteric vein | Portal | Peritoneum |  |  | Right paracolic gutter |
| Right paracolic gutter peritoneum | Superior mesenteric vein → portal vein | Superior mesenteric vein | Portal | Peritoneum |  | Mesothelial | Right paracolic gutter |
| Gallbladder peritoneum | Cystic vein → portal vein | Cystic vein | Portal | Upper abdomen |  | Mesothelial | Right subphrenic space |
| Right diaphragmatic dome | Right inferior phrenic vein → IVC | Inferior phrenic vein | Inferior vena cava | Upper abdomen |  | Muscular | Right subphrenic space |
| Right diaphragmatic dome peritoneum | Inferior phrenic vein → IVC | Inferior phrenic vein | Inferior vena cava | Upper abdomen |  | Mesothelial | Right subphrenic space |
| Right diaphragmatic peritoneum | Right inferior phrenic vein → IVC | Inferior phrenic vein | Inferior vena cava | Upper abdomen |  | Mesothelial | Right subphrenic space |
| Morrison’s pouch | Right inferior phrenic vein → IVC / portal via hepatic veins | Inferior phrenic vein | Portal | Upper abdomen |  | Mesothelial | Subhepatic recess (Morrison's pouch) |
| Morrison’s pouch II | Right inferior phrenic vein → IVC / portal via hepatic veins | Inferior phrenic vein | Portal | Upper abdomen |  | Mesothelial | Subhepatic recess (Morrison's pouch) |
| Morrison’s pouch peritoneum | Right inferior phrenic vein → IVC / portal via hepatic veins | Inferior phrenic vein | Portal | Upper abdomen |  | Mesothelial | Subhepatic recess (Morrison's pouch) |

**Supplementary Table 2**: Grouping of tumor locations, assessed during cytoreductive surgery of high-grade serous ovarian cancer patients, into oncological relevant compartments in correlation to pre-operative spread of circulating tumor cells.

| **Compartment** | **correlation** | **conf.low** | **conf.high** | **p.value** | **adj.p.value** | **Significance** |
| --- | --- | --- | --- | --- | --- | --- |
| Lymph nodes | 0.4104 | 0.1653 | 0.6077 | 0.0017 | 0.0151 | * |
| Peritoneum | 0.1778 | -0.0893 | 0.421 | 0.1899 | 0.6924 |  |
| Retroperitoneum | 0.1627 | -0.1046 | 0.4082 | 0.2308 | 0.6924 |  |
| Lower abdomen | -0.1164 | -0.368 | 0.1511 | 0.3929 | 0.8801 |  |
| Bowel | -0.072 | -0.3287 | 0.1946 | 0.598 | 0.8801 |  |
| Abdominal wall | -0.0624 | -0.3201 | 0.2038 | 0.6477 | 0.8801 |  |
| Pelvis | 0.0534 | -0.2125 | 0.3119 | 0.6957 | 0.8801 |  |
| Mesentery | -0.0378 | -0.2977 | 0.2274 | 0.7823 | 0.8801 |  |
| Upper abdomen | -0.0131 | -0.275 | 0.2507 | 0.9239 | 0.9239 |  |

**Supplementary Table 3**: Grouping of tumor locations, assessed during cytoreductive surgery of high-grade serous ovarian cancer patients into the affected organ’s type of tissue in correlation to pre-operative spread of circulating tumor cells.

| **Tissue type** | **correlation** | **conf.low** | **conf.high** | **p.value** | **adj.p.value** | **Significance** |
| --- | --- | --- | --- | --- | --- | --- |
| Lymphatic | 0.4104 | 0.1653 | 0.6077 | 0.0017 | 0.0134 | * |
| Muscular | 0.1373 | -0.1303 | 0.3862 | 0.313 | 0.8478 |  |
| Epithelial | -0.1272 | -0.3775 | 0.1404 | 0.3501 | 0.8478 |  |
| Mesothelial | 0.109 | -0.1584 | 0.3615 | 0.4239 | 0.8478 |  |
| Subcutaneous | -0.0715 | -0.3282 | 0.195 | 0.6004 | 0.9348 |  |
| Connective | 0.0459 | -0.2197 | 0.3051 | 0.7369 | 0.9348 |  |
| Adipose | -0.0155 | -0.2773 | 0.2484 | 0.9098 | 0.9348 |  |
| Vascular | -0.0112 | -0.2733 | 0.2525 | 0.9348 | 0.9348 |  |

**Supplementary Table 4**: Oncology relevant nodal basins, removed during cytoreductive surgery of high-grade serous ovarian cancer patients in correlation to pre-operative spread of circulating tumor cells.

| **Nodal basins** | **correlation** | **conf.low** | **conf.high** | **p.value** | **adj.p.value** | **Significance** |
| --- | --- | --- | --- | --- | --- | --- |
| External iliac | 0.5094 | 0.2846 | 0.6811 | 0.0001 | 0.0005 | *** |
| Paraaortic / Caval | 0.4354 | 0.1948 | 0.6266 | 0.0008 | 0.0021 | ** |
| Pelvic | 0.4357 | 0.1952 | 0.6268 | 0.0008 | 0.0021 | ** |
| Common iliac | 0.3122 | 0.0537 | 0.5315 | 0.0192 | 0.0278 | * |
| Extra-abdominal | 0.3081 | 0.0492 | 0.5282 | 0.0209 | 0.0278 | * |
| Upper abdominal | 0.3081 | 0.0492 | 0.5282 | 0.0209 | 0.0278 | * |
| Inguinal | -0.059 | -0.317 | 0.2071 | 0.6658 | 0.6658 |  |
| Internal iliac | -0.059 | -0.317 | 0.2071 | 0.6658 | 0.6658 |  |

**Supplementary Table 5**: Peritoneal fluid dynamics zones in correlation to pre-operative spread of circulating tumor cells in high-grade serous ovarian cancer patients.

| **Zone** | **correlation** | **conf.low** | **conf.high** | **p.value** | **adj.p.value** |
| --- | --- | --- | --- | --- | --- |
| Right paracolic gutter | 0.1638 | -0.1036 | 0.4091 | 0.2277 | 0.8838 |
| Left subphrenic space | 0.1417 | -0.1259 | 0.3901 | 0.2975 | 0.8838 |
| Subhepatic recess (Morrison's pouch) | 0.1323 | -0.1353 | 0.3819 | 0.331 | 0.8838 |
| Lesser sac (omental bursa) | -0.1059 | -0.3588 | 0.1615 | 0.4374 | 0.8838 |
| Anterior abdominal peritoneum | -0.0839 | -0.3393 | 0.1831 | 0.5388 | 0.8838 |
| Right subphrenic space | 0.0737 | -0.1929 | 0.3302 | 0.5892 | 0.8838 |
| Left paracolic gutter | -0.0349 | -0.2951 | 0.2301 | 0.7983 | 0.9177 |
| Pelvic cavity (Douglas pouch) | 0.0319 | -0.233 | 0.2923 | 0.8157 | 0.9177 |
| Inframesocolic compartment | -0.0128 | -0.2748 | 0.2509 | 0.9251 | 0.9251 |
